# Deciphering the Glycan Preference of Bacterial Lectins by Glycan Array and Molecular Docking with Validation by Microcalorimetry and Crystallography

**DOI:** 10.1371/journal.pone.0071149

**Published:** 2013-08-19

**Authors:** Jeremie Topin, Julie Arnaud, Anita Sarkar, Aymeric Audfray, Emilie Gillon, Serge Perez, Helene Jamet, Annabelle Varrot, Anne Imberty, Aline Thomas

**Affiliations:** 1 CERMAV- Centre national de la recherche scientifique UPR5301 (affiliated to Université Joseph Fourier and ICMG), BP53, 38041 Grenoble, France; 2 Département de Chimie Moléculaire, UMR- Centre national de la recherche scientifique 5250 & ICMG FR 2607, Université Joseph Fourier, BP 53, 38041 Grenoble, France; French National Centre for Scientific Research - Université de Toulouse, France

## Abstract

Recent advances in glycobiology revealed the essential role of lectins for deciphering the glycocode by specific recognition of carbohydrates. Integrated multiscale approaches are needed for characterizing lectin specificity: combining on one hand high-throughput analysis by glycan array experiments and systematic molecular docking of oligosaccharide libraries and on the other hand detailed analysis of the lectin/oligosaccharide interaction by x-ray crystallography, microcalorimetry and free energy calculations. The lectins LecB from *Pseudomonas aeruginosa* and BambL from *Burkholderia ambifaria* are part of the virulence factors used by the pathogenic bacteria to invade the targeted host. These two lectins are not related but both recognize fucosylated oligosaccharides such as the histo-blood group oligosaccharides of the ABH(O) and Lewis epitopes. The specificities were characterized using semi-quantitative data from glycan array and analyzed by molecular docking with the Glide software. Reliable prediction of protein/oligosaccharide structures could be obtained as validated by existing crystal structures of complexes. Additionally, the crystal structure of BambL/Lewis x was determined at 1.6 Å resolution, which confirms that Lewis x has to adopt a high-energy conformation so as to bind to this lectin. Free energies of binding were calculated using a procedure combining the Glide docking protocol followed by free energy rescoring with the Prime/Molecular Mechanics Generalized Born Surface Area (MM-GBSA) method. The calculated data were in reasonable agreement with experimental free energies of binding obtained by titration microcalorimetry. The established predictive protocol is proposed to rationalize large sets of data such as glycan arrays and to help in lead discovery projects based on such high throughput technology.

## Introduction

Interaction between lectins, or other carbohydrate binding proteins, and carbohydrates play important roles in many biological and pathological processes. In the last years, high throughput analysis of glycome or availability of glycan microarrays [1], [2] revolutionized the glycobiology field. Integrated approaches are now needed for combining data arising from investigations performed at different scales. The wealth of information obtained from glycan array experiments and available in databases has to be combined with experimental data that describe the structure and affinity of single lectin binding sites. Molecular modeling can also be performed with different levels of sophistication and represents an excellent tool for integrating and rationalizing data obtained from different methods.

Carbohydrate binding proteins from pathogenic bacteria, such as lectins, adhesins or toxins are interesting models since, as a result of co-evolution, they have the capacity to specifically recognize complex oligosaccharides present on host tissues [3]–[5]. Among the human glycoconjugates that can be targeted by bacterial lectins, the A, B, and H antigens are complex fucosylated oligosaccharides present on endothelial cells and erythrocytes of all individuals of blood group A, B, or O, respectively [6]. In addition, Lewis epitopes, that are also fucosylated oligosaccharides, depend on the Lewis genotype of the individuals. The biological role of the ABO and Lewis histo-blood group systems remains to be elucidated, but since the 80s, several studies have pointed out correlations between the repartition of phenotype in population and the susceptibility to diseases [7], [8]. The most cited examples are the individuals with O phenotype who present higher susceptibility towards severe forms of cholera [9], [10] and gastroenteritis caused by Norwalk virus [11]. For individuals with secretor phenotype, blood-group related epitopes are also present in lung mucus. The nature of the oligosaccharides present in airways depends not only on ABO, Lewis and secretor genotypes but also on long-term inflammatory diseases such as chronic bronchitis and cystic fibrosis (CF). More particularly, fucosylated glycoconjugates, which are present in higher quantity in mucins [12] and N-glycans [13] of lungs of CF patients, appear to be the target for lectins from pathogenic bacteria that are responsible for morbidity and mortality in CF patients.

**Figure 1:**
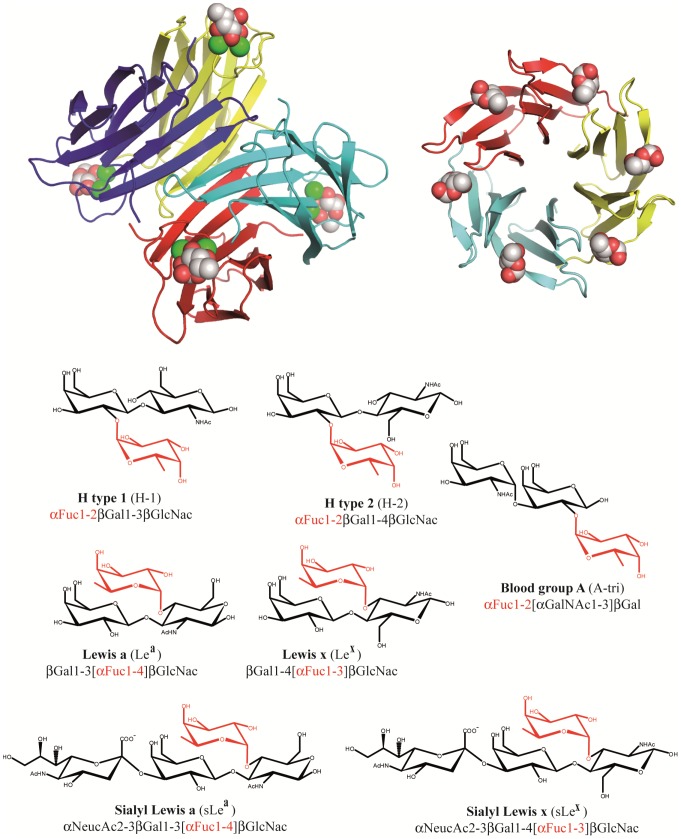
Representation of the bacterial lectins and oligosaccharides used in the docking calculations. Top: Graphical representation of the two bacterial lectins. From left to right LecB/fucose and BambL/fucose complexes have been adapted from PDB 1GZT and 3ZW2 respectively. The fucose ligand and calcium atoms are displayed as spheres. Bottom: Schematic representation and sequences of fucosylated oligosaccharides.

Soluble lectins with high affinity for human fucosylated oligosaccharides have been identified in *Pseudomonas aeruginosa* and bacteria from the *Burkholderia cepacia* complex such as *B. cenocepacia* and *B. ambifaria* [14]–[17]. LecB (also named PA-IIL) from *P. aeruginosa* is a tetrameric protein that displays an unusually strong micromolar affinity for l-fucose in a tight binding site which requires two Ca^2+^ ions [18], [19]. BambL from *B. ambifaria* is a trimeric lectin arranged in a β-propeller fold with two similar binding sites per monomer, resulting in a hexameric arrangement of the fucose binding sites [14]. Both lectins were shown to bind to a large variety of fucosylated oligosaccharides with LecB having higher affinity for the Lewis a epitope [20] and BambL for the H-type 2 epitope [14]. An illustration of the two fucose-binding lectins is displayed in Figure 1.

Bacteria utilize therefore a variety of multimer topologies and different fucose binding sites with various specificities towards fucosylated human histo-blood group oligosaccharides and molecular modeling is an approach of choice for rationalizing the observed specificities. Even though molecular docking calculations are routinely performed on small organic compounds, docking oligosaccharides is still a challenging task due to the constraints of ring shape (fucose preferentially adopts a ^1^C_4_ conformation), the occurrence of CH-π interactions between aromatic amino-acids and the C-H bonds of sugars, and the role of peculiar stereo-electronic effect such as anomeric and exo-anomeric effects which are not handled in standard molecular mechanics algorithms [21], [22]. Nevertheless, conformational analysis of histo-blood group epitopes demonstrated that these oligosaccharides can adopt a limited number of well defined conformations [23], [24]. These are available in the BIOLIGO database (http://bioligo.cermav.cnrs.fr/) that contains three-dimensional structures of bioactive oligosaccharides generated using the SWEET-II [25] and POLYS [26] builders altogether with Shape a conformational search engine based on a genetic algorithm coupled to the MM3 force-field [27]. Molecular docking studies of fucose and fucose-containing oligosaccharides have been published using a variety of computational approaches, in protein targets such as human lectins [28], [29], antibodies [30], [31] or virus capsid proteins [32]. Molecular docking calculations of fucose and methyl-fucoside in bacterial lectins were also reported even though the presence of calcium ions in some of the binding sites was computationally challenging [33]–[37].

With increasing availability of glycan arrays which allow for rapid deciphering of lectin specificity [1], the need for efficient protocols for structural rationalization of data is urgent. In this paper, we propose a docking strategy based on the use of starting oligosaccharides conformations derived from the BIOLIGO database. The models are validated by comparison with available crystal structures, including the newly solved structure of the BambL/Lewis x (Le^x^) complex. An additional step of free energy calculations using the Molecular Mechanics Generalized Born Surface Area (MM-GBSA) provides theoretical affinities that can be compared to thermodynamic data determined from titration microcalorimetry experiments.

## Materials and Methods

### Experimental section

#### Material

Human histo-blood group oligosaccharides were obtained from Elicityl (Crolles, France). The two bacterial lectins were produced recombinantly in *Escherichia coli* as previously described [14], [19].

#### Glycan array

Purified LecB sample was labeled with Alexa Fluor 488-TFP (Invitrogen, CA) according to manufacturer's instructions and re-purified on a D-Salt polyacrylamide desalting column (Pierce, Rockford IL). Alexa-labeled protein was used for glycan array screening with standard procedure of the Core H of the Consortium for Functional Glycomics (http://www.functionalglycomics.org, Emory University, Atlanta, GA, USA). The labeled lectin was assayed on the CFG glycan array version 4.1 comprising 465 natural and synthetic glycans and the data were analyzed in a dose-dependent manner as previously described [38] at 10, 1 and 0.1 µg ml^−1^ of LecB (PA-IIL) and 1, 0.2 and 0.05 µg ml^−1^ of BambL dissolved in 20 mM HEPES, 140 mM NaCl, 5 mM CaCl_2_, pH 7.5.

#### Isothermal Titration Calorimetry (ITC)

Recombinant lyophilized LecB was dissolved in buffer (20 mM Tris/HCl pH 7.5, NaCl 150 mM with 0.03 mM CaCl_2_). Protein concentration was checked by measurement of A_280_ by using a theoretical molar extinction coefficient of 6990 M^−1^ cm^−1^. Carbohydrate ligands (H-type 1, H-type 2 and blood group A trisaccharides, sialyl Lewis x and sialyl Lewis a tetrasaccharides) were dissolved in the same buffer, and loaded in the injection syringe. ITC was performed with an ITC200 microcalorimeter (MicroCal Inc.). Lectin solution was placed (150 µM) in the 200 µl sample cell, at 25°C. Titration was performed with 20 of 2 µl injections of carbohydrate ligands (1.3 to 2.0 mM) every 300 s. Data were fitted with MicroCal Origin 7 software, according to standard procedures. Fitted data yielded the stoichiometry (n), the association contant (K_a_) and the enthalpy of binding (ΔH). Other thermodynamic parameters (i.e. changes in free energy, ΔG, and entropy, ΔS) were calculated from the equation ΔG = ΔH-TΔS = −RT *ln* K_a_ in which T is the absolute temperature and R = 8.314 J mol^−1^ K^−1^. Two independent titrations were performed for each ligand tested.

#### Crystallogenesis, data collection and structure determination

Crystals of BambL complexed with Le^x^ tetrasaccharide were obtained by the hanging-drop vapor diffusion method using 2 µl drops containing a 50∶50 (v/v) mix of protein and reservoir solution at 19°C. Lyophilized protein was dissolved in Tris/HCl buffer pH 7.5, NaCl 150 mM at 1 mM and incubated for 1 h with 5 mM of sugar at room temperature prior to co-crystallization. Reservoir solution consisted of 200 mM Trisodium citrate, 100 mM sodium acetate pH 5.0 and 28% Peg 6K. 10% ethylene glycol was added for cryoprotection prior mounting in a cryoloop and flash-freezing in liquid nitrogen. Diffraction data were collected at 100K, at the European Synchrotron Radiation Facility (Grenoble, France) on ID14-EH4 beamline using an ADSC Q4 CCD detector. The data were processed using MOSFLM [39] and scaled and all further computing was performed using the CCP4 suite unless otherwise stated [40]. Data quality statistics are summarized in Table S1. Molecular replacement was used to obtain the initial phases with the program PHASER [41] and one monomer of the native structure of BambL (PDB ID: 3ZW0) as searching model. The structure was refined by restrained maximum-likelihood refinement using REFMAC 5.5 [42] with 5% of the data retained for cross-validation interspersed with model building and electron density inspection in Coot [43]. The incorporation of the sugar moieties was performed after inspection of the the 2mFo–DFc and mFo–DFc weighted maps. Water molecules were introduced automatically using Coot and inspected manually. The coordinates have been deposited in the Protein Data Bank under code 3ZW1. All figures were drawn with The PyMOL Molecular Graphics System (Schrödinger, LLC).

### Modeling section

**Figure 2:**
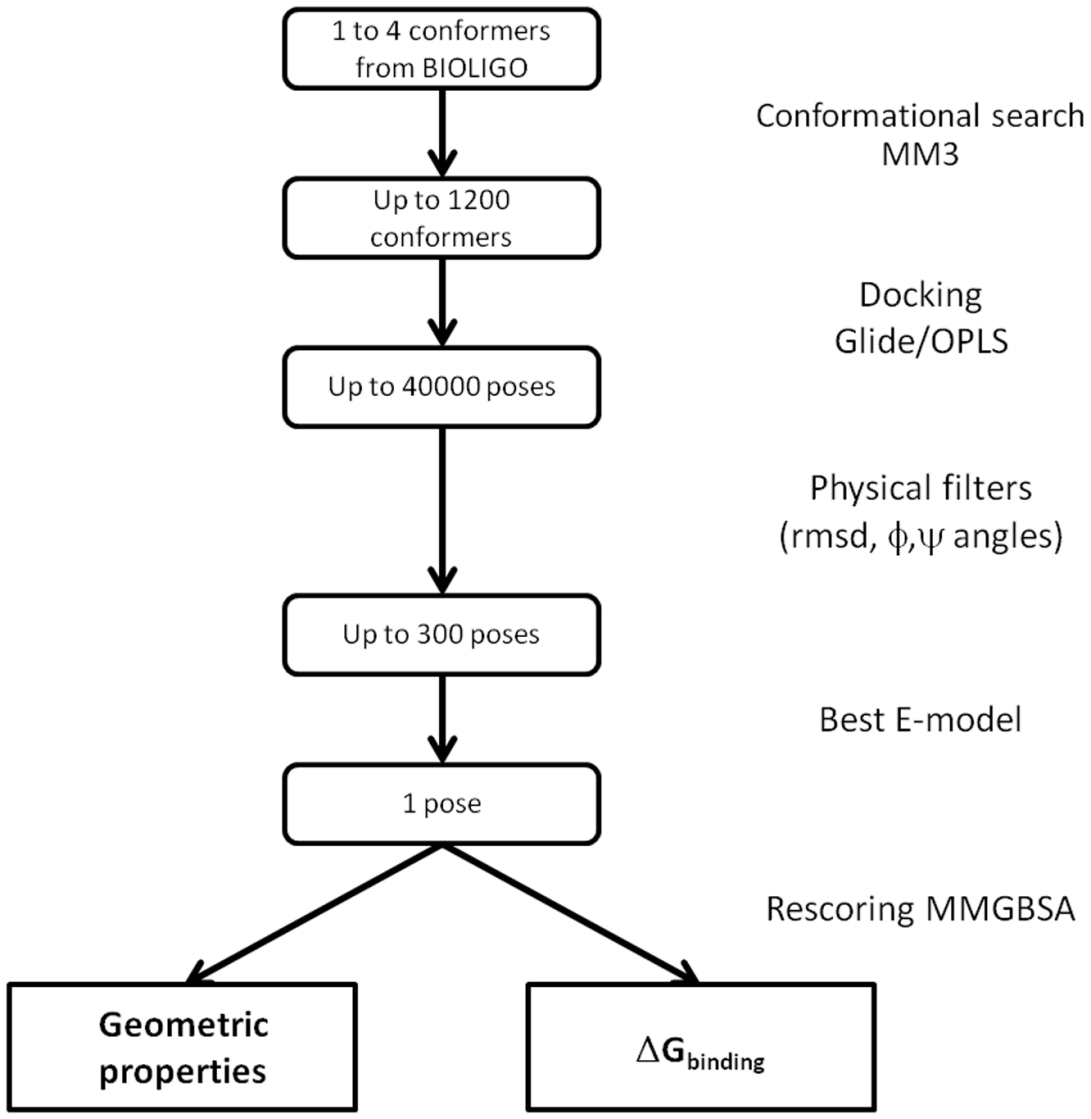
Flowchart of the docking/analysis protocol.

All calculations were performed using the Schrödinger Suite of programs through the Maestro graphical interface (http://www.schrodinger.com, Schrödinger LLC, New York). The overall docking protocol and filtering procedure are schematized in Figure 2.

#### Preparation of the ligands

Models of the seven fucose-containing oligosaccharides displayed in Figure 1 have been built. Between two and four different conformations were considered for each structure, first one by extracting coordinates from the crystal structures of the complex when available and others one by selecting low energy conformations from the BIOLIGO database (http://glyco3d.cermav.cnrs.fr/bioligo). The dihedral angle values of the glycosidic linkages of the starting conformers are provided in Table S2.

#### Preparation of the proteins

The crystal structures of two lectin/carbohydrate complexes, LecB/Lewis a (PDB ID: 1W8H) [20] and BambL/H type 2 (PDB ID: 3ZZV) [14] were taken from the Protein Data Bank [44]. The four binding sites of LecB contain a conserved water molecule in close interaction with the protein and the fucose moiety. This structural water molecule is also found in other LecB crystal structures, and was thus explicitly included in all calculations. Since the lectin BambL possesses two similar binding sites per monomer, only the intra-monomeric site was selected for the calculations. The hydrogen atoms were added assessing a pH of 7. The histidine residues were treated as neutral. The selection of the histidine enantiomer and the orientation of asparagine and glutamine side-chains were performed so as to maximize hydrogen bond patterns. The partial atomic charges derived from the OPLS-2005 [45] were assigned to the ligands, calcium ions and protein atoms. Finally, energy minimizations were performed using the Impref utility of the Schrödinger package and the OPLS-2005 force-field.

#### Docking Calculations

The docking program Glide [46], [47] has been shown to give good performances for docking carbohydrates [37]. Rigid-receptor docking calculations were run in standard precision mode, with the OPLS-2005 force field, while the ligand was considered as flexible. The shape and physico-chemical properties of the binding sites were mapped onto cubic grid of 24 Å side centered on the ligand. During the grid generation, a scaling of van der Waals radii of 1 for atoms with partial charges less than 0.25 e was used. Prior to docking, each conformer was submitted to an extensive conformational search protocol using the MM3 force field to generate several hundreds of conformers. Parameters for van der Waals radii were scaled by 0.80 for atoms with partial charges less than 0.15. Ring conformational sampling was not allowed in order to maintain the starting conformations, particularly the ^1^C_4_ conformation of the l-fucose ring; no other constraints were introduced. Ligand poses were clustered with a RMS tolerance of 1.3 Å. A maximum of 10,000 poses could be retained for the initial docking phase (i.e. up to 40,000 with four starting oligosaccharide conformations), and were energy minimized.

A hierarchical post-docking filtering procedure was developed to take into account the specific nature and requirements of the studied lectins and of the low energy oligosaccharide structures. Otherwise, the combination of the OPLS-2005 force-field coupled to Glide could generate oligosaccharide structures corresponding to high energy conformers. Poses were first filtered according to the RMSD between heavy atoms of the fucose residue of the poses and of the fucose present in the crystal starting structure (threshold of 1 Å). Additional geometrical filters were also developed to reject distorted geometries. Poses having Φ and Ψ glycosidic dihedral angles outside the known low energy regions (±10°) were rejected. These low-energy regions were based on the previously calculated adiabatic maps of disaccharides [23] available from the Glyco3D site (http://www.cermav.cnrs.fr/Glyco3D). Finally, the poses having an energetically unfavorable cis conformation of the amide bond in N-acetyl groups [48] were discarded. Between 2 to 300 binding poses for each starting conformer were finally retained from this procedure, and were all visually inspected. The docked solutions were ranked using Emodel, a composite scoring function, available in the Schrödinger Suite and that is a combination of GlideScore, of the ligand receptor molecular mechanics interaction energy, and of the ligand strain energy (Friesner et al. 2004). This score, which better takes into account electrostatic and van der Waals energy, is particularly well suited for comparing conformers but it should be used together with another scoring function while comparing different species.

#### MMGBSA rescoring

The binding free energy of each best docked poses was estimated using the local optimization feature in Prime software (Schrödinger) based on the molecular mechanics (MM) OPLS-2005 force field and the Generalized Born Surface Accessible (GBSA) continuum model. Such MM-GBSA rescoring protocol has proved to be successful [49]. The binding free energy of each ligand ΔG_bind_ is described as the difference in the sums of MM energy, GBSA solvation energy (including surface area energy SA) of the complex on one hand and of the free ligand and free protein on the other hand. Strain energies were calculated for both the protein and the ligand. This feature is directly implemented in the Schrödinger suite and consists in quantifying the difference in energy between the free and the complex state of both the ligand and the protein after energy minimization. The energy minimization of the protein, was performed using Prime by allowing the side-chains within 10 Å of the ligand to move, in order to avoid large structure deviation.

## Results

### Specificity analysis by glycan array

**Figure 3:**
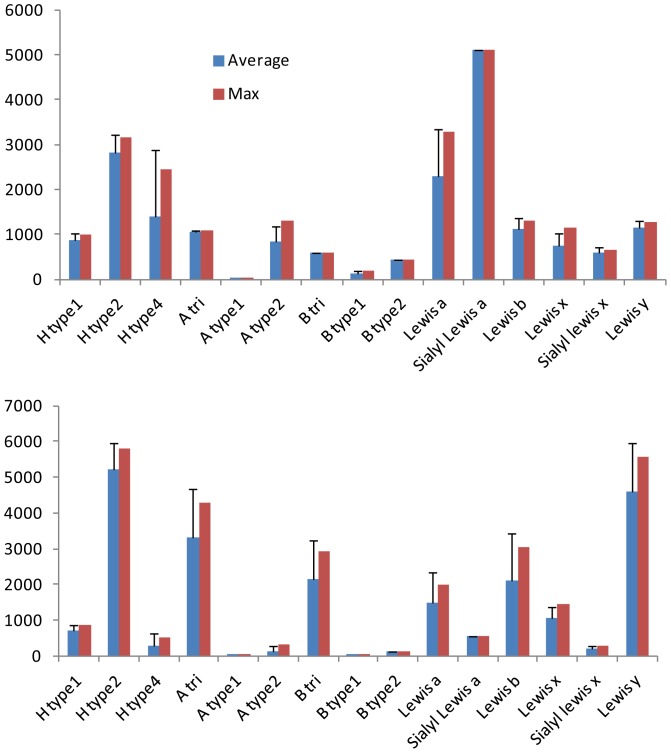
Selected data from the glycan array v4.1 performed on two bacterial lectins. Binding is represented as fluorescent intensity obtained with LecB (top panel) and BambL (bottom panel) both at 0.2 µg.mL^−1^ and labeled with Alexa 488. Blue bar: average value with standard deviation over 5 experiments at same concentration, red bar: maximum response observed.

The fine specificity of the two bacterial lectins was analyzed using glycan array v4.1 from the Consortium for Functional Glycomics that contains 465 glycoconjugates (http://www.functionalglycomics.org). The overall glycan array data for BambL was described previously [14] and those for LecB are available from the CFG website. Briefly, BambL only attaches to the oligosaccharides presenting at least one fucose residue, whereas LecB displays also some weak affinity towards mannose-containing glycans. In order to set up a comparison with the docking results, binding data were analyzed by selecting only glycans having one fucosylated epitope at the non-reducing position. This resulted in data for 53 glycans with two lectins at three concentrations (see Tables S3 and S4). Each of these 53 glycans could be assigned to one of the 15 human epitopes. The different concentrations gave very consistent data, and only the results corresponding to one lectin concentration are given in Figure 3.

Very different binding patterns were observed for the two bacterial lectins. The glycan array of LecB confirmed its preference for Lewis a (Le^a^) epitopes with highest apparent affinity for sialyl Lewis a (sLe^a^). H type 2 is also a strong binder, whereas other fucosylated glycans resulted in moderate binding, except A type 1 and B type 1 that were not recognized. Despite the fact that BambL was described as a fucose-binding lectin with broad specificity [14], the present analysis indicated its preference for H type 2 and Lewis y (Le^y^) oligosaccharides.

### Docking of fucosylated oligosaccharides in bacterial lectins

Among the 14 oligosaccharides analyzed by glycan array, seven were selected for the docking approaches based on their biological relevance and diversity (Figure 1). Lewis b and Lewis y were not included since the occurrence of two fucose residues per oligosaccharides would complicate the interpretation of docking results.

#### Validation of the molecular docking protocol on the LecB/Le^a^ complex

The docking protocol with selection of adequate scoring function was setup so as to reproduce the binding mode of Le^a^ in LecB (rmsd ligand of 0.5 Å), which is the most difficult to handle due to the presence of the two calcium ions, treated with a net +2 charge in a non-polarizable force field. Solution NMR experiments [24] and theoretical studies [23] have proposed an almost rigid conformation for this branched trisaccharide with parallel piling (pseudo-stacking) of the galactose and fucose rings (conf 1 in Table S2). This low energy conformation is also present in the crystal structure of LecB/Le^a^ complex [20]. Nevertheless, recent NMR studies concluded for some conformation flexibility [50]. 130 binding poses were obtained for Le^a^ in the active site of LecB. The scoring function GlideScore did not rank the crystal structure conformation as the best pose. This could be corrected by the use of the composite scoring function called Emodel [46], which combines the binding affinity predicted by GlideScore and the internal strain energy of the ligand (see Figure S1). The Emodel score was thus used to rank all the oligosaccharide binding poses in both lectins.

#### Docking of fucosylated oligosaccharides in LecB from *P. aeruginosa*

The seven oligosaccharides of interest were docked within the LecB binding site. As imposed with the filtering protocol, the fucose moiety always coordinated the two calcium ions through oxygen atoms O-2, O-3 and O-4. The shallow shape of the binding site allowed the oligosaccharides to adopt a variety of conformations. Since the present study aims at a medium throughput rationalization of glycan array data, only the pose with the best Emodel score is further discussed. It should be noted that this pose did not always originate from calculations starting with the lowest energy model from BIOLIGO database, i.e. conformer 1 in Table S2. In 75% of the complexes, the best pose was obtained starting from other conformations taken from BIOLIGO database.

**Figure 4:**
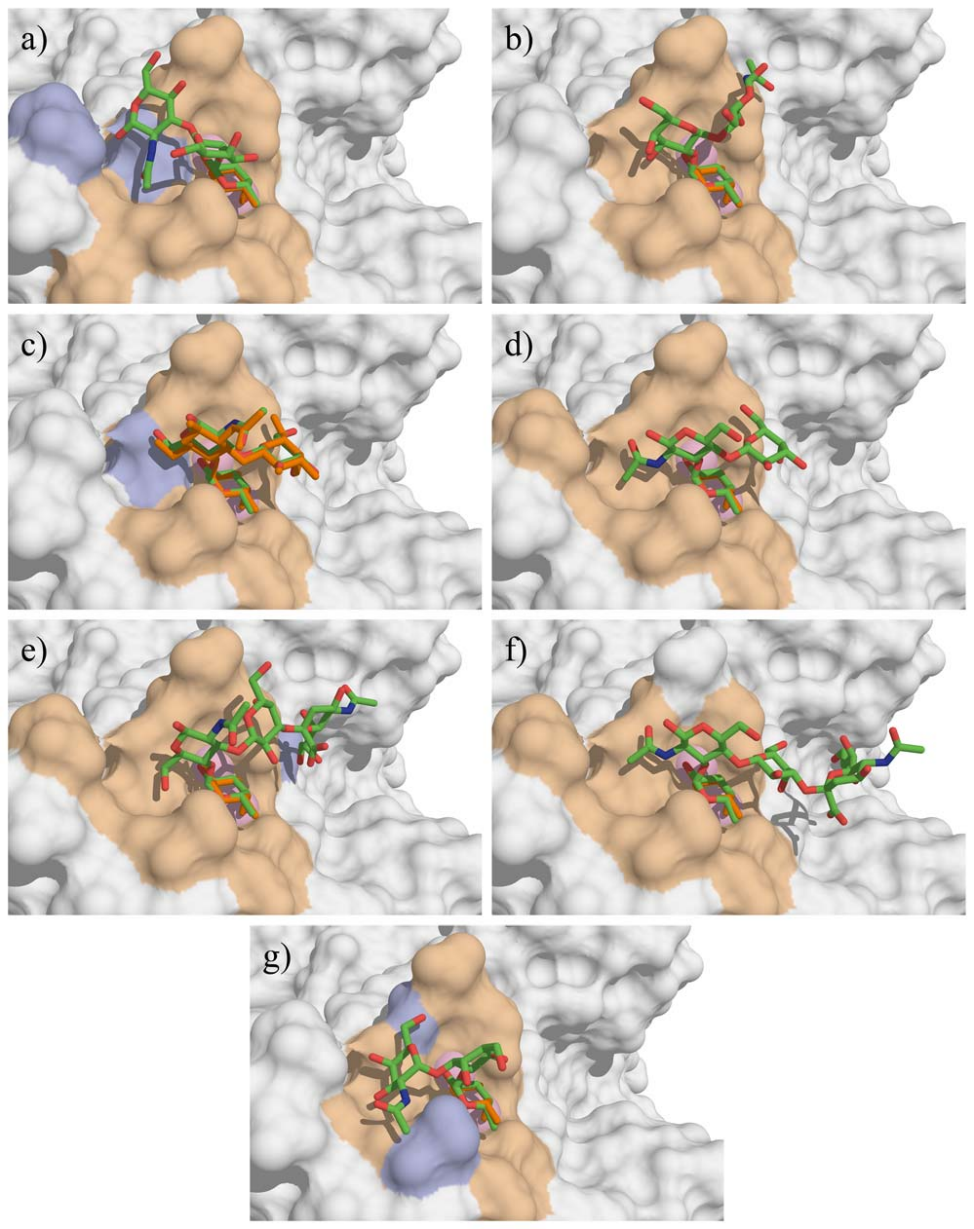
Docking of the oligosaccharides in LecB binding site. a) H type 1, b) H type 2, c) Le^a^, d) Le^x^, e) sLe^a^ f) sLe^x^ and g) A-tri. The docked oligosaccharides are represented as sticks (carbon, oxygen and nitrogen atoms are colored green, red and blue respectively) and the ones from crystal structures are colored orange. Calcium ions are represented as pink spheres. The protein accessible surface is colored in beige for residues comprised within a sphere of 4 Å around the ligand and in blue for residues involved in hydrogen bond with ligand residues (except fucose).

**Figure 5:**
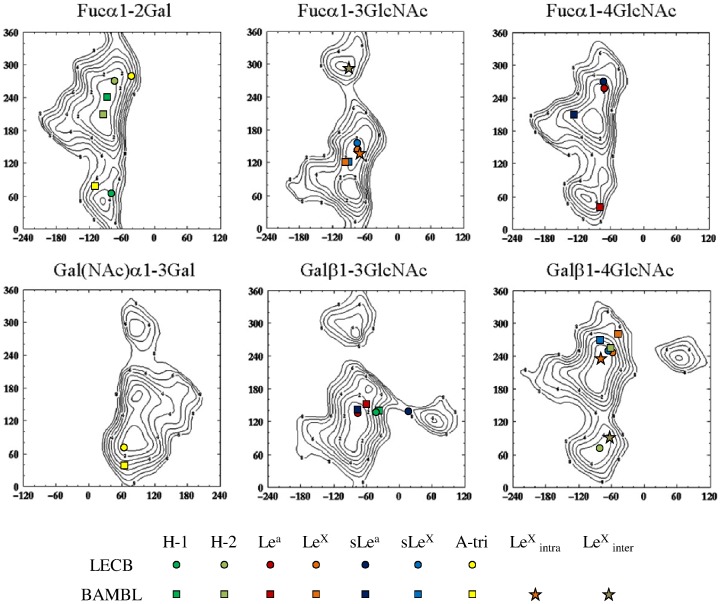
Energy maps and docked conformations. Isoenergy contrours (1 kcal/mole) are represented as a function of Φ (x-axis) and Ψ (y-axis)torsion angles for all glycosidic linkages of interest taken from Glyco3D (http://glyco3d.cermav.cnrs.fr/) with superimposition of oligosaccharide conformations derived from docking (circles and squares) or protein crystallography (stars). Torsion angles are defined as Φ = θ(O5-C1-O1-Cx) and Ψ = θ(C1-O1-C′x-C′x+1).

The best poses are displayed in Figure 4 and their structural analysis is given in Table 1. Despite their structural similarities, H type 1 and H type 2 trisaccharides presented very different conformations (Figure 4a and 4b). The difference in αFuc1-2Gal linkage conformation is illustrated in Figure 5 whereas the values of Φ and Ψ torsion angles are reported on the previously calculated energy maps [23] that give an estimation of the available conformational space for each glycosidic linkage. For both trisaccharides, one of the two glycosidic linkages adopted the lowest energy conformation, while the other one lied in a secondary minimum corresponding to a 120° difference in Ψ angle compared to low energy region. H-type 1 established two hydrogen bonds but since these involved reducing O1 of GlcNAc, they will not occur when GlcNAc is conjugated such as on glycan arrays. The only predicted hydrogen bonds involved the anomeric oxygen to GlcNAc. The slightly higher affinity of LecB for H type 2 trisaccharide is therefore proposed to originate from hydrophobic contact involving N-acetyl group of sugar and CH_2_ group of Asp99 side chain.

**Table 1:** Characterization of the best poses of the fucosylated oligosaccharides in the binding site of LecB.

	Φ	Ψ	Φ	Ψ	Hydrogen bonds	Hydrophobic contacts	Glide Emodel	ΔG_MMGBSA_
**H-1**	αFuc1-2Gal		βGal1-3GlcNAc		GlcNAc.O1…R72.NH2	GlcNAc.CH3…G24	−97.4	−125.9
	−74	65	−47	160	GlcNAc.O1…D96.O	GlcNAC.CH3…T98		
**H-2**	αFuc1-2Gal		βGal1-4GlcNAc				−92.4	−108.8
	−80	272	−81	61		GlcNAc.CH3…D99		
**Le^a^**	αFuc1-4GlcNAc		βGal1-3GlcNAc				−85.8	−127.0
	−77	260	−68	131	GlcNAc.O6…D96.O			
**Le^x^**	αFuc1-3GlcNAc		βGal1-4GlcNAc			GlcNAc.CH3…V69	−88.6	−120.1
	−68	147	−66	254				
**sLe^a^**	αFuc1-4GlcNAc		βGal1-3GlcNAc		Sia.O8…E86.OE		−91.3	−129.7
	−76	272	11	147				
**sLe^x^**	αFuc1-3GlcNAc		βGal1-4GlcNAc			GlcNAc.CH3…V69	−87.9	−118.9
	−72	148	−72	256				
**A-tri**	αFuc1-2Gal		αGalNAc1-3Gal		GalNAc.O4…R72.NH2		−90.8	−111.9
	−48	272	63	71	GalNAc.O4…D96.O			
					Gal.O4…S23.OG			

The glycosydic dihedrals Φ and Ψ are indicated in degrees. The hydrogen bonds and hydrophobic contacts between the ligands and residues located less than 4 Å of any ligand atom (except fucose) are listed. The associated Glide Emodel score and binding free energy are given in kcal/mol.

Both Le^a^ and Le^x^ trisaccharides bound in their lowest energy conformation (Figure 4c and 4d), with stacking of the galactose ring above the fucose one. The docking confirmed that, as proposed from the crystal structure [20], the preference of LecB for binding to Le^a^ trisaccharide originated from an additional hydrogen bond between O6 of GlcNAc and Asp96. The additional neuraminic acid residue present in sLe^a^ tetrasaccharide established one additional hydrogen bond between glycerol side chain and Glu86 acidic group, which correlates well with the high affinity observed on the glycan array. The blood group A trisaccharide (A-tri) also established several hydrogen bonds with the protein residues, but the presence of αGalNAc forces the αFuc1-2Gal linkage in a strained conformation, that may explain the observed lowest affinity.

#### Docking of fucosylated oligosaccharides in BambL from *B. ambifaria*

**Figure 6:**
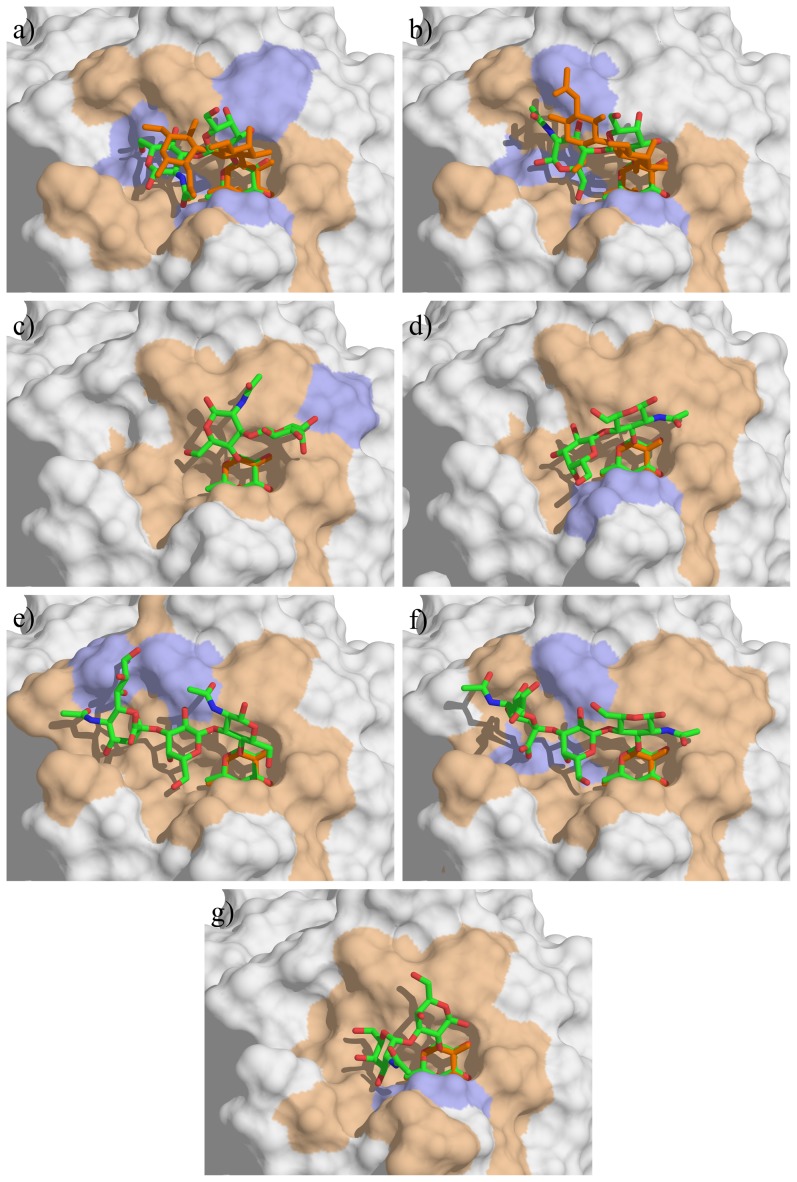
Docking of the oligosaccharides in BambL binding site. a) H type 1, b) H type 2, c) Le^a^, d) Le^x^, e) sLe^a^ f) sLe^x^ and g) A-tri. The color codes are the same as in Figure 4.

The resulting best docked solutions obtained with BambL according to the Emodel score are represented in Figure 6 and their structures characterized in Table 2. The active-site in BambL is deeper than in LecB, constraining the oligosaccharide conformation and allowing more contacts between the ligands and the surrounding residues. The αFuc1-2Gal linkage of H type 1 and H type 2 trisaccharides maintained its lowest energy conformation upon binding, resulting in same localization of galactose residues for both trisaccharides, and several hydrogen bonds are established in addition to those involving fucose. However, the βGal1-4GlcNAc linkage of H type 2 is bound in the lowest energy conformation, whereas the βGal1-4GlcNAc linkage of H type 1 is forced in a higher energy one. This may be correlated to the preference of the lectin for H-type 2. It should be noted that both H-type 1 and H-type 2 poses are in good agreement with the crystal structures of their complexes with BambL [14], with rmsd for ligands of 1.6 and 2.6 Å, respectively. The αFuc1-2Gal linkage in blood group A adopted the secondary minimum conformation with large shift in Ψ value, and the αGalNAc establishes several contacts with the protein surface.

**Table 2:** Characterization of the best poses of the fucosylated oligosaccharides in the binding site of BambL.

	Φ	Ψ	Φ	Ψ	H bond	Hydroph	Glide Emodel	ΔG_MMGBSA_
**H-1**	αFuc1-2Gal		βGal1-3GlcNAc		Gal.O3…Y35.OH	GlcNAc.CH3…G74	−82.0	−72.4
	−93	238.	−41	134	GlcNAc.O…W74.NE1	GlcNAc.CH3…W76		
					GlcNAc.O6…S13.OG			
					GlcNAc.O6…W8.NE1			
**H-2**	αFuc1-2Gal		βGal1-4GlcNAc		GlcNAc.O1…W8.NE1	GlcNAc.C6…W74	−80.8	−83.1
	−106	219	−59	249	GlcNAc.N…S13.OH			
					GlcNAc.O3…D30.O			
					GlcNAc.O6…W74.NE1			
**Le^a^**	αFuc1-4GlcNAc		βGal1-3GlcNAc		Gal.O6…T36.O	GlcNAc.CH3…Y35	−70.4	−81.8
	−917	45	−59	153				
**Le^x^**	αFuc1-3GlcNAc		βGal1-4GlcNAc		Gal.O4…W74.NE1	GlcNAc.CH3…A38	−66.7	−71.6
	−104	120	−48	285	Gal.O6…G76.O			
**sLe^a^**	αFuc1-4GlcNAc		βGal1-3GlcNAc		Gal.O2…D30.OD	Gal.C6…W74	−93.8	−96.8
	−127	214	−74	136	Sia.O8…D30.O	Gal.C6…V57		
					Sia.CO…T11.N	Sia.C2…W8		
**sLe^x^**	αFuc1-3GlcNAc		βGal1-4GlcNAc		Gal.O2…TD30.OD	GlcNAc.CH3…Y35	−90.9	−65.7
	−113	105	−73	271	Sia.O1…W8.NE1	Gal.C6…V57		
					Sia.CO…T11.OG			
**A-tri**	αFuc1-2Gal		αGalNAc1-3Gal		GalNAc.O4…W74.NE1	GalNAc.C4…V57	−70.7	−83.9
	−111	69	57	42		GalNAc.C6…W8		

The glycosydic dihedrals Φ and Ψ are in degrees. The hydrogen bonds and hydrophobic contacts between the ligands and residues located less than 4 Å of any ligand atom (excepted the fucose) are listed. The associated Glide Emodel and binding free energy are given in kcal/mol.

The docking of Lewis oligosaccharides is of special interest. Indeed, since Trp74 is stacked against the fucose hydrophobic face in the binding site, the solution conformation of Le^a^ and Le^x^, with galactose piling up on fucose, cannot be accommodated. Nevertheless, BambL binds significantly to Lewis oligosaccharides on the glycan arrays (Figure 3). Our docking calculations predicted that this “closed” conformation has to open, for pushing the galactose on the surface of the binding site. For Le^a^ trisaccharide this is performed by a conformational change of the αFuc1-4GlcNAc linkage that occupies the secondary minimum with Ψ conformation close to 60° (Figure 5) resulting in a “twist shape”. For Le^x^, each glycosidic linkage remained in the main energy well, but with significant deviation (up to 30°) for the global minimum resulting in a “semi-open” shape. This conformation occurred for both sLe^a^ and sLe^x^ but the distortion of linkages seems to be unfavorable since both tetrasaccharides are not efficiently recognized by BambL on glycan array.

### Crystal structure of BambL/Lewis x complex and comparison with docking

#### Overall structure

BambL was cocrystallised with the Le^x^ tetrasaccharide and crystals belonging to the P2_1_3 spacegroup with cell parameters a = b = c = 81.4 Å and α = β = γ = 90° appeared after 5 days. The complex structure was solved by molecular replacement and two molecules were found in the asymmetric unit which led to a solvent content of 46.5%. Each belongs to a trimer generated by applying crystallographic three-fold symmetry (Figure S2). The final model was refined in the resolution range between 40.7 and 1.6 Å to R and R_free_ values of 15.3 and 18.9 respectively. It is composed of all 87 amino acids in each monomer and the N-terminal methionine of chain A was found oxidized. No conformational changes are observed between the two molecules or when compared with the native structure. Only slight movements are present at the level of some surface loops.

#### Oligosaccharide binding site

The initial electronic density maps revealed clearly the presence of the fucose moiety in the four available binding sites (two per monomers) and of a variable number of additional sugars depending on the binding site. In the intermolecular site of A molecule and in the intramolecular site of B molecule only the αFuc1-3GlcNAc moiety could be fitted in the density whereas the other residues are too disordered to be located. In both cases, the disaccharide adopted an unusual conformation with of Ψ torsion angle (approx −60°) corresponding to a secondary minimum in the low energy map (Figure 5). This particular conformation brings the N-acetyl group of GlcNAc in a groove on the protein surface.

**Figure 7:**
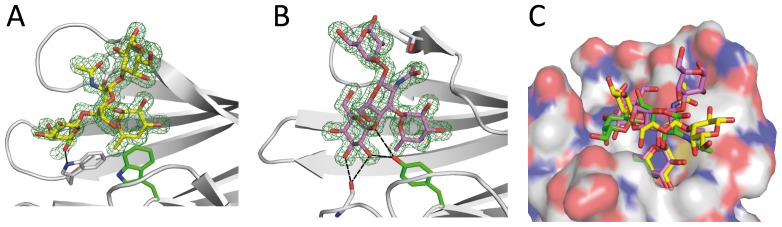
Crystallographic structure of BambL in complex with Le^x^ tetrasaccharide. (A) Intramonomeric and (B) intermonomeric sites. Tetrasaccharide and key amino acids are represented as sticks, hydrogen bond to non-fucose carbohydrate residues as dotted lines; 2mF_0_-Df_c_ electron density map contoured at 1σ is shown ad green mesh. (C) Superimposition of Le^x^ tetrasaccharide from crystal intramonomeric (yellow) and intermonomeric (pink) sites and Le^x^ trisaccharide from docking (green).

In the two other sites, clear electron density was observed for the whole Le^x^ tetrasaccharide (Figure 7). In the intermolecular site of B, the reducing galactose displayed some mobility according to B-factor and density. It only makes interactions mediated by water molecules with the symmetric molecule through it O6 and O4 hydroxyl groups. All hydroxyls of the non-reducing galactose are involved in hydrogen bonds either direct or mediated by solvent. The O4 oxygen interacts with Thr36 and the O6 with Asp75 via a water molecule. All other contacts are established with a neighboring protein unit generated by packing symmetry. The O6 atom of the GlcNAc moiety is hydrogen bonded to Arg27and the O7 and N1 are water bridged to Arg81 and Asp75, respectively. The αFuc1-3GlcNAc conformation corresponds to the low energy region of the map (Φ = −91°, Ψ = 149°), but the βGal1-4GlcNAc adopts a secondary minimum (Φ = −51°, Ψ = 81°) as displayed in Figure 5. In the intramolecular site of A molecule, the reducing galactose only interacts with the symmetry-generated protein mainly via direct contacts with O1, O2, O4 and O5 atoms. For the non-reducing galactose, only the O4 hydroxyl makes a weak contact with Asp30 through solvent. At the level of the GlcNAc moiety, the pyranose ring is distorted to a ^3,O^B boat conformation so that the N acetyl group stacks onto Tyr35 ring. This could not happen in the intermolecular site since there is a threonine at this position and therefore no possibility of steric clashes. Both αFuc1-3GlcNAc and βGal1-4GlcNAc linkages belong to the main energy minimum, but the overall conformation is very unusual and does not present the classical stacking between fucose and galactose ring due to the distorsion of the GlcNAc ring.

Comparison between the two conformations observed in the crystal structure and the best pose obtained by docking is represented in Figure 7c Although the predicted pose is not identical to any of the ones in the crystal structure (rmsd values of 3.15 and 2.17 Å), the docking correctly predicted the general distorted shape of Lewis x in the binding site of BambL

### Thermodynamic of binding by titration microcalorimetry

Glycan array data provide evaluation of the specificity of the lectins, but for precise measurement of affinity, and comparison with calculated binding energies, titration calorimetry is the most relevant approach. Thermodynamic data for BambL interacting with oligosaccharides were determined previously [14] therefore only LecB was assayed by ITC (Table 3). The seven fucosylated oligosaccharides present affinities for LecB in the micromolar range and a typical thermogram is presented in Figure S3. The solution data are in good agreement with glycan array data (Figure 3) with highest affinity for Le^a^ and sLe^a^ (although with no preference for this latest one). H type 2 is slightly better ligand than H type 1, and A trisaccharide is the weaker binder among the tested oligosaccharides. BambL ITC data presented more differences with glycan array maybe due to stronger surface effects due to the topology of the lectin. In solution, blood group A and H type 2 oligosaccharides are the highest affinity ligands, and the sialylated oligosaccharides the weakest ones.

**Table 3:** Titration microcalorimetry data for the interaction between bacterial lectins and fucosylated ligands.

	LecB			BambL		
Ligand	K_D_ (µM)	ΔG (kcal/mol)	ΔH (kcal/mol)	K_D_ (µM)	ΔG (kcal/mol)	ΔH (kcal/mol)
H type 1	1.39	−8.0	−7.7	*26.1* b	*−6.3*	*−4.2*
H type 2	*0.48*	*−8.6*	*−9.4*	*7.5* b	*−7.0*	*−10.6*
Le^a^	0.21^a^	−9.1	−8.4	*18.2* b	*−6.5*	*−6.9*
Le^x^	3.44^a^	−7.4	−5.3	*34.8* b	*−6.1*	*−9.3*
sLe^x^	5.7	−7.2	−5.6	39.5	−6.0	−16.4
sLe^a^	0.29	−8.9	−9.3	61.3	−5.7	−15.4
A tri	10.3	−6.8	−6.0	0.46^b^	−8.6	−12.7

All data have been measured at least twice and standard deviations are below 15%. The numbers in italics correspond to experimental data obtained with longer oligosaccharides (i.e. one additional glucose on reducing end).

a from [20],

b from [14].

### Energy calculation and comparison with experimental data

Attempts to correlate the different scoring functions available within Glide with the experimental free energy of binding did not yield any satisfactory results. The binding free energy of each best docked poses was therefore rescored using MM-GBSA approach. The obtained values are listed in the last column of Table 1 and Table 2 for LecB and BambL, respectively.

#### Energy calculations for LecB/oligosaccharide complexes

**Figure 8:**
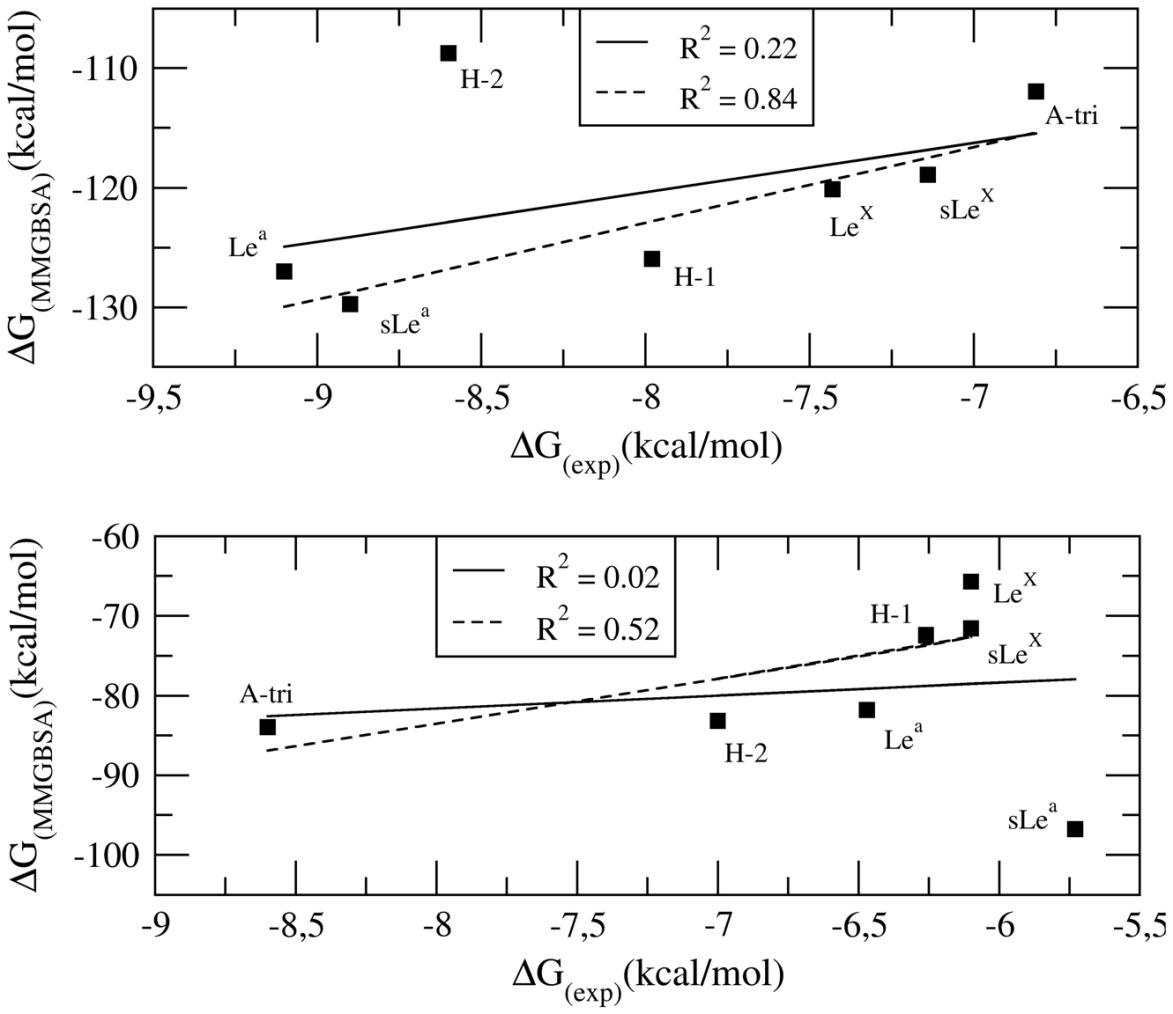
Correlations between experimental binding free energies of binding measured from titration microcalorimetry and theoretical ones calculated using MM-GBSA. Top: LecB, bottom: BambL (bottom). Calculations have been performed with all data (black lines) or with omission of H-2 for LecB or sLea for BambL, respectively.

Le^a^ and sLe^a^ oligosaccharides gave the lowest MM-GBSA energies for binding to LecB in agreement with both glycan array and ITC data. The other predicted affinities were ranked correctly, with the exception of H-type 2 that is a clear false negative. Correlation has been attempted by plotting the theoretical binding energies versus the experimental free energy of binding obtained from ITC (Figure 8). The range for the ΔG_(MMGBSA)_ energies is larger than the one for ITC energies. This difference could arise from the lack of terms describing enthalpy/entropy compensation in the MM-GBSA calculation. The overall agreement was not very good (R^2^ = 0.22) but improved significantly (R^2^ = 0.84) when omitting the H type 2 trisaccharide that has wrongly been predicted to be a poor ligand.

#### Energy calculations for BambL/oligosaccharide complexes

While sLe^x^ was correctly predicted to be a poor ligand for BambL, the isomer sLe^a^ displayed the best calculated affinity with MM-GBSA, which classified it as false positive since the experimental affinity is weak. Apart from these two charged tetrasaccharides, the binding energies of all oligosaccharides were in a limited range, varying from −71.6 kcal/mol for Le^x^ to −83.1 kcal/mol for blood group A trisaccharide. This is in rather good agreement with ITC data that did not show a large range of affinities for BambL binding to non-charged fucosylated oligosaccharides, with preference for A blood group trisaccharide (Figure 8). When excluding sLe^a^ as a false positive, a correlation factor of 0.52 is obtained between theoretical and experimental free energies of binding.

## Discussion and Conclusion

The development of glycan arrays allowed studying the fine specificities of glycan-binding proteins. Even though some authors have developed systematic analyzes of glycan array data to identify the lectin specificities [51], [52], there is a lack of automatic methods to extract structural information from glycan arrays. A docking protocol of bioactive oligosaccharides that would be common to fucose-binding lectins was investigated in this study, so as to structurally rationalize large amounts of experimental affinities, such as those arising from glycan array experiments. Among the different protocols evaluated, the refined molecular docking procedure combines Glide in standard precision for generating the poses and the Emodel scoring function for ranking them. Starting from several conformers derived from the BIOLIGO database greatly enhanced the conformational sampling, as up to four conformers for each molecule were introduced in the docking procedure. Using this approach, we could predict the conformation of oligosaccharides upon lectin binding and analyze the network of stabilizing contacts established. This helped in rationalizing the preference of LecB for Lewis a oligosaccharides.

Interestingly, the approach was also very efficient in predicting twisted or strained conformations of Lewis oligosaccharides in interaction with BambL. Because of their branched structures on position 3 and 4 of GlcNAc (Figure 1), Le^a^ and Le^x^ are unusually rigid oligosaccharides. They have been described as mainly rigid, with only one low energy conformation by the early work of Lemieux and Bock [24], later confirmed by X-ray structure [53], theoretical calculations [23] and several NMR studies [54], [55]. Only the αFuc1-2 linkage of some Le^a^ analogs was proposed to display some flexibility [50]. This conformational stability has been proposed to be important to understand the recognition by antibodies [56], and to our knowledge, no other conformation has been observed in available crystal structures of lectin/Lewis oligosaccharides complexes (see: http://lectin3d.cermav.cnrs.fr). BambL is therefore the first protein that binds Lewis oligosaccharide in conformations that are very different than their solution shape. The particular localization of one tryptophan residue excludes the low energy conformation of Lewis oligosaccharide from the binding site. The strong stacking interaction that fucose establishes with this tryptophan residue in the binding site of RSL, a lectin closely related to BambL, was recently quantified [57]. Solving the crystal structure of BambL/Lewis x complex demonstrated that indeed, the fucose is bound with stacking to Trp, but that either the adjacent glycosidic linkages or the GlcNAc ring are distorted in order to adjust.

As reported in the literature [49], [58], [59], the MM-GBSA rescoring calculations strongly increase the correlation between theoretical data and quantitative thermodynamic data. Even though lectin-oligosaccharide interactions are generally enthalpy-driven (Table 3), incorporation of solvation energy term, including polar component and surface area accessibility, is necessary to obtain reliable order of binding energies of oligosaccharides due to the some entropy contribution in binding,. The best correlation was achieved for LecB, for which the parameters used in the calculations were developed. The procedure selected for LecB was rather successful when applied to BambL, demonstrating that the procedure is valid for binding sites with different characteristics (local charges, solvent exposure…). Deviations between glycan array data and MM-GBSA calculations are expected, and among the numerous sources of discrepancies one could cite the fact that the glycans are methylated, and conformationally restricted as they are immobilized on a surface, usually through covalent interactions. On the contrary, the oligosaccharides used in the microcalorimetry experiments, have a free hydroxyl in C1, and can dynamically exchange between an open form, and a closed form, either with an axial or equatorial anomeric position, while only the equatorial structures were considered in the calculations. Also, one should keep in mind that trisaccharides were used for docking studies while experimental affinities were mostly performed on their tetrasaccharide analogs.

Even though molecular dynamics simulations in explicit water environment is better suited for estimating the binding free energies, the combined use of database of oligosaccharide conformations and fast docking procedure appears as a medium-throughput screening approach for the analysis of glycan array data. To our knowledge, this is the first study combining theoretical calculations and glycan array data in the perspective of dealing with a huge flow of information such as the one coming in this Omics era.

## Supporting Information

Figure S1 **Best docked solution of Le^a^ in the binding site of LecB. Le^a^ is represented in color coded sticks (cyan, red and blue for carbon, oxygen and nitrogen atoms, respectively).** The calcium ions are drawn as pink spheres. The docked pose is nearly identical to the crystallographic conformation of Le^a^ (in orange sticks), except the hydroxyl moiety on C6 of the galactose residue.
(PDF)

Figure S2 **Crystal structure of BambL complexed with Le^x^ trisaccharide.** The asymmetric unit is represented in blue and magenta and the corresponding β-propellers obtained by 3-fold crystalline symmetry operations are represented in light blue and light pink.
(PDF)

Figure S3 **Thermogram (top) and titration curve (bottom) obtained from the titration of LecB (150 µM) by sialyl Lewis a (1.3 mM).** Fitting procedure was performed using a one site model.
(PDF)

Table S1 **Details of collection data and statistics.**
(PDF)

Table S2 **Glycosidic linkage conformations for all starting models of oligosaccharides.** The Φ and Ψ torsion angles for a glycosidic 1—x linkage are described as Φ = O5-C1-O1-C′_x_ and Ψ = C1-O1-C′_x_-C′_x+1_.
(PDF)

Table S3 **Binding intensities (FU) for labeled LecB protein with glycan array chips v4.1 from the consortium for functional glycomics.** Full data is available on the web site (http://www.functionalglycomics.org/).
(PDF)

Table S4 **Binding intensities (FU) for labeled BambL protein with glycan array chips v4.1 from the consortium for functional glycomics.** Full data is available on the web site (http://www.functionalglycomics.org/).
(PDF)
