# Supplementary material for: Deciphering the Glycan Preference of Bacterial Lectins by Glycan Array and Molecular Docking with Validation by Microcalorimetry and Crystallography
Source: PLoS One. 2013 Aug 19;8(8):e71149. doi: 10.1371/journal.pone.0071149 (PMC3747263; doi:10.1371/journal.pone.0071149)
Supplement: Figure S1 — Best docked solution of Lea in the binding site of LecB. Lea is represented in color coded sticks (cyan, red and blue for carbon, oxygen and nitrogen atoms, respectively). The calcium ions are drawn as pink spheres. The docked pose is nearly identical to the crystallographic conformation of Lea (in orange sticks), except the hydroxyl moiety on C6 of the galactose residue. (PDF) [file pone.0071149.s001.pdf]

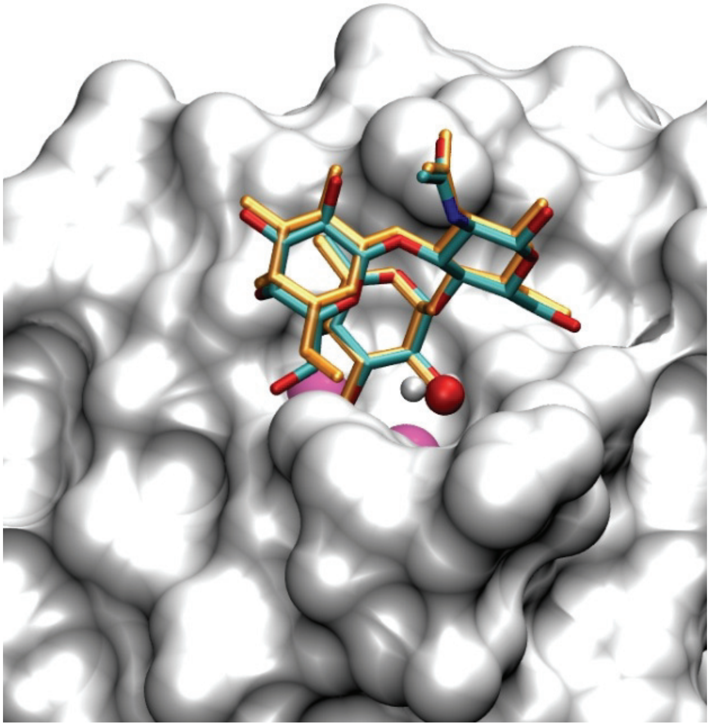

Figure S1: Best docked solution of Le[a] in the binding site of LecB. Le[a] is represented in color coded sticks (cyan, red and blue for carbon, oxygen and nitrogen atoms, respectively). The calcium ions are drawn as pink spheres. The docked pose is nearly identical to the crystallographic conformation of Le[a] (in orange sticks), except the hydroxyl moiety on C6 of the galactose residue.
