# Supplementary material for: Deciphering the Glycan Preference of Bacterial Lectins by Glycan Array and Molecular Docking with Validation by Microcalorimetry and Crystallography
Source: PLoS One. 2013 Aug 19;8(8):e71149. doi: 10.1371/journal.pone.0071149 (PMC3747263; doi:10.1371/journal.pone.0071149)
Supplement: Figure S2 — Crystal structure of BambL complexed with Lex trisaccharide. The asymmetric unit is represented in blue and magenta and the corresponding β-propellers obtained by 3-fold crystalline symmetry operations are represented in light blue and light pink. (PDF) [file pone.0071149.s002.pdf]

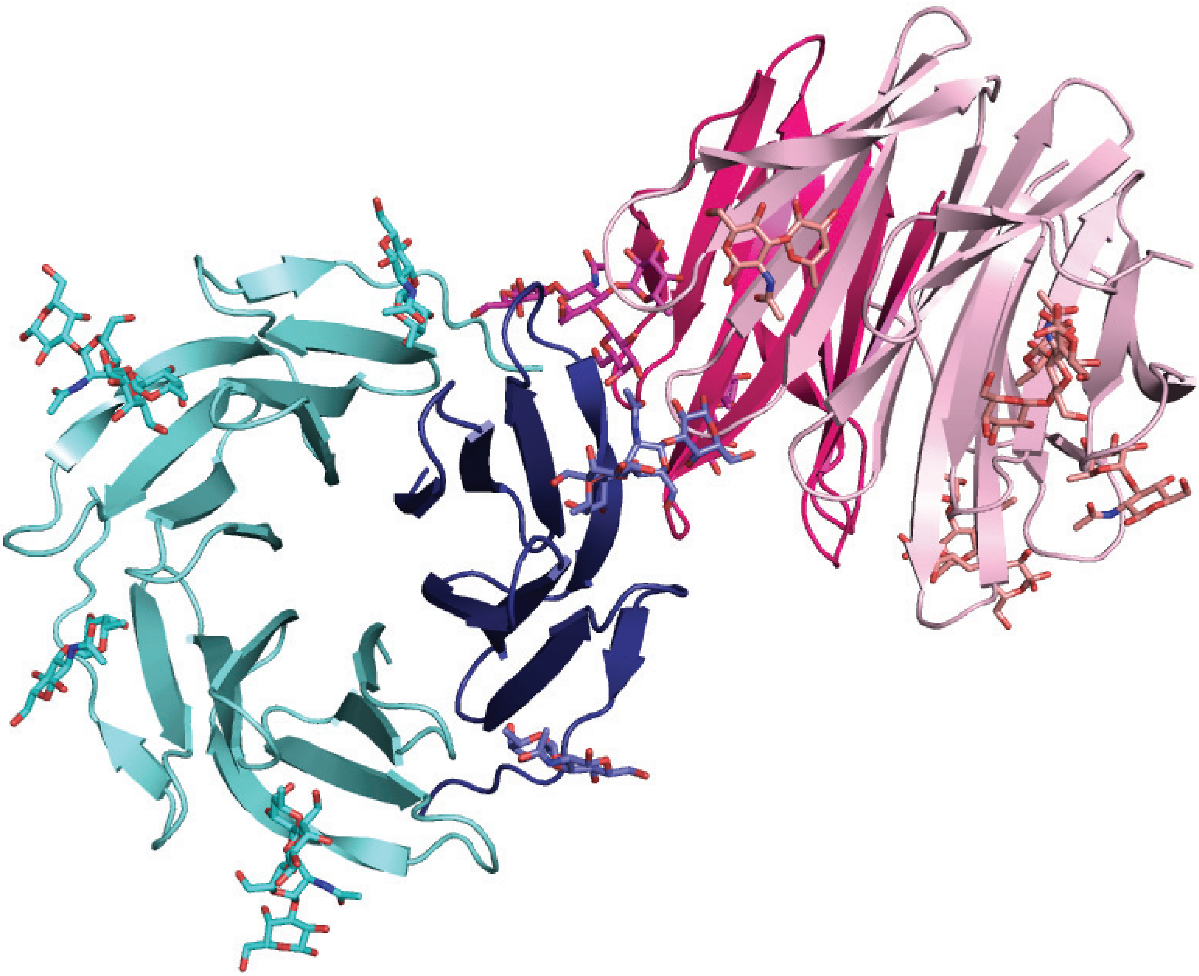

Figure S2: Crystal structure of BambL complexed with Le^x trisaccharide. The asymmetric unit is represented in blue and magenta and the corresponding β-propellers obtained by 3-fold crystalline symmetry operations are represented in light blue and light pink.
