# Supplementary material for: Deciphering the Glycan Preference of Bacterial Lectins by Glycan Array and Molecular Docking with Validation by Microcalorimetry and Crystallography
Source: PLoS One. 2013 Aug 19;8(8):e71149. doi: 10.1371/journal.pone.0071149 (PMC3747263; doi:10.1371/journal.pone.0071149)
Supplement: Figure S3 — Thermogram (top) and titration curve (bottom) obtained from the titration of LecB (150 µM) by sialyl Lewis a (1.3 mM). Fitting procedure was performed using a one site model. (PDF) [file pone.0071149.s003.pdf]

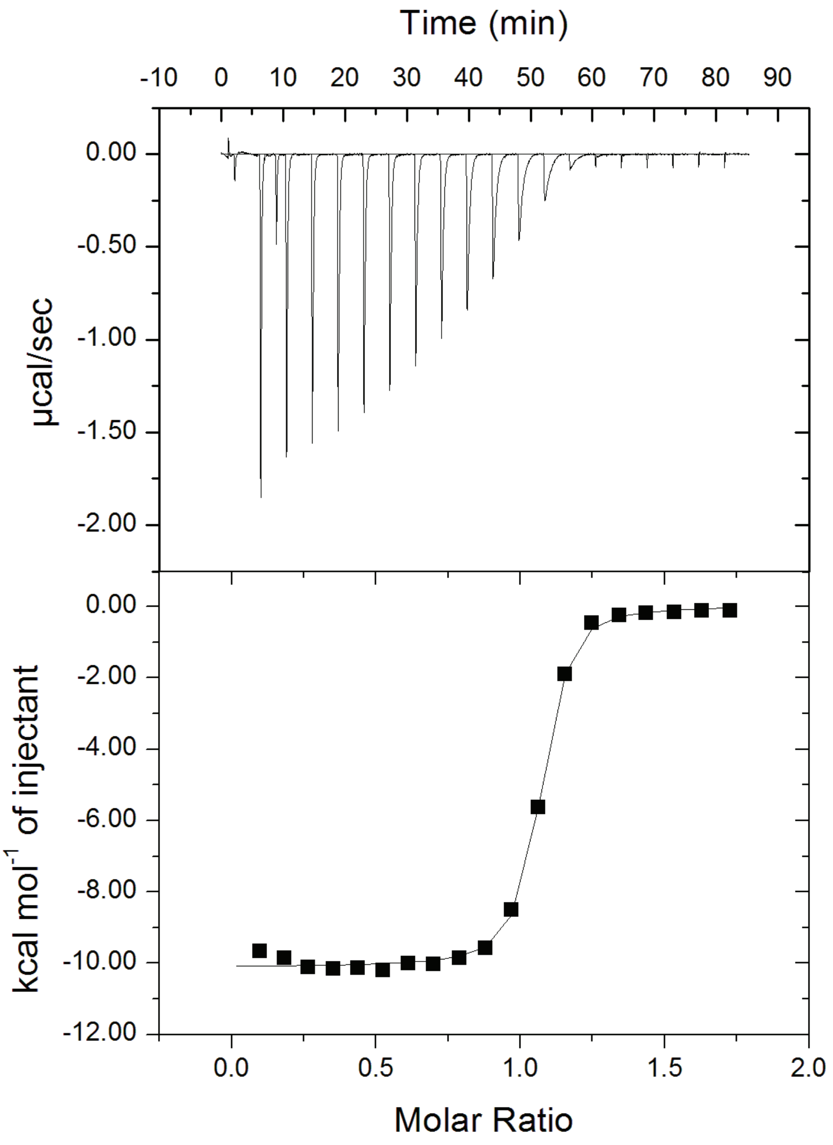

Figure S3: Thermogram (top) and titration curve (bottom) obtained from the titration of LecB (150 µM) by sialyl Lewis a (1.3 mM). Fitting procedure was performed using a one site model.
