# Supplementary material for: Deciphering the Glycan Preference of Bacterial Lectins by Glycan Array and Molecular Docking with Validation by Microcalorimetry and Crystallography
Source: PLoS One. 2013 Aug 19;8(8):e71149. doi: 10.1371/journal.pone.0071149 (PMC3747263; doi:10.1371/journal.pone.0071149)
Supplement: Table S1 — Details of collection data and statistics. (PDF) [file pone.0071149.s004.pdf]

Table S1: Details of collection data and statistics.

**Protein**	**BambL/leX**
**Data collection**	
Wavelength (Å)	1.140
Space group	P 2_1 3
Cell dimensions a, b, c (Å)	81.4, 81.4, 81.4
Resolution (outer shell) (Å)	40.72-1.60 (1.69-1.60)
Measured/Unique reflections	21930/1180
Average muliplicity (outer shell)	6.2 (3.4)
Rmerge (outer shell)	0.04 (0.1)
Completeness (%) (outer shell)	96.2 (82.4)
Mean I/oI (outer shell)	26.6 (7.9)
Wilson B	17.4
**Refinement**	
Rcryst/Rfree	15.5/18.9
r.m.s.d. bonds (Å)	0.016
r.m.s.d. angles (°)	1.71
r.m.s.d. chiral ($Å^3$)	0.10
Protein atoms	1357
Bfac ($Å^2$)	14.4
Ligand atoms	156
Bfac ($Å^2$)	21.0
Water molecules	237
Bfac ($Å^2$)	27.72
PDB code	3ZW1
