# Supplementary material for: Deciphering the Glycan Preference of Bacterial Lectins by Glycan Array and Molecular Docking with Validation by Microcalorimetry and Crystallography
Source: PLoS One. 2013 Aug 19;8(8):e71149. doi: 10.1371/journal.pone.0071149 (PMC3747263; doi:10.1371/journal.pone.0071149)
Supplement: Table S2 — Glycosidic linkage conformations for all starting models of oligosaccharides. The Φ and Ψ torsion angles for a glycosidic 1—x linkage are described as Φ = O5-C1-O1-C′x and Ψ = C1-O1-C′x-C′x+1. (PDF) [file pone.0071149.s005.pdf]

Table S2: Glycosidic linkage conformations for all starting models of oligosaccharides.

The Φ / Ψ torsion angles for a glycosidic 1—x linkage are described as Φ = O5-C1-O1-C'$_x$ and Ψ= C1-O1-C'$_x$-C'$_{x+1}$

dihedral angle	conf	H-1		H-2		A-tri		Le^x		sLe^x		Le^a		sLe^a	
		ϕ	ψ	ϕ	ψ	ϕ	ψ	ϕ	ψ	ϕ	ψ	ϕ	ψ	ϕ	ψ
Fucα1-2Gal	1	-80	264	-79	266	-95	193								
	2	-97	192	-95	191	-69	273								
	3	-104	194	-76	271	-89	184								
Fucα1-3GlcNAc	1							-78	150	-77	145				
	2							-151	92	-77	145				
Fucα1-4GlcNAc	1											-79	262	-80	259
	2											-104	193	-79	260
	3													-79	260
	4													-93	51.8
Galβ1-3GlcNAc	1	-74	137									-76	142	-69	134
	2	-89	67									-64	172	-71	135
	3	-64	169											-71	134
	4													-64	159
Galβ1-4GlcNAc	1			-76	247			-75	-104	-73	252				
	2			-88	182			-77	-102	-71	360				
	3			-80	64										
GalNAcα1-3Gal	1					72	78								
	2					71	68								
	3					90	175								
