# Supplementary material for: Deciphering the Glycan Preference of Bacterial Lectins by Glycan Array and Molecular Docking with Validation by Microcalorimetry and Crystallography
Source: PLoS One. 2013 Aug 19;8(8):e71149. doi: 10.1371/journal.pone.0071149 (PMC3747263; doi:10.1371/journal.pone.0071149)
Supplement: Table S3 — Binding intensities (FU) for labeled LecB protein with glycan array chips v4.1 from the consortium for functional glycomics. Full data is available on the web site (http://www.functionalglycomics.org/). (PDF) [file pone.0071149.s006.pdf]

Table S3: Binding intensities (FU) for labeled LecB protein with glycan array chips v4.1 from the consortium for functional glycomics. Full data is available on the web site (http://www.functionalglycomics.org/)

		10 µg/ml	1 µg/ml	0.1 µg/ml
	Glycan Structure	Average	Average	Average
H-di	Fucα1-2Galβ-Sp8	9118	1947	115
H_type1	Fucα1-2Galβ1-3GlcNAcβ1-3Galβ1-4Glcβ-Sp8	5510	1003	20
H-type1	Fucα1-2Galβ1-3GlcNAcβ1-3Galβ1-4Glcβ-Sp10	3834	853	21
H-type1	Fucα1-2Galβ1-3GlcNAcβ-Sp0	4526	902	29
H-type1	Fucα1-2Galβ1-3GlcNAcβ-Sp8	3393	636	12
H-type1	Fucα1-2Galβ1-3GlcNAcβ1-3(Galβ1-4GlcNAcβ1-6)Galβ1-4Glc-Sp21	6054	1009	51
H-type2	Fucα1-2Galβ1-4GlcNAcβ1-3Galβ1-4GlcNAcβ-Sp0	13494	2887	284
H-type2	Fucα1-2Galβ1-4GlcNAcβ1-3Galβ1-4GlcNAcβ1-3Galβ1-4GlcNAcβ-Sp0	15404	3167	199
H-type2	Fucα1-2Galβ1-4GlcNAcβ-Sp0	13127	2953	309
H-type2	Fucα1-2Galβ1-4GlcNAcβ-Sp8	10059	2235	132
H-type3	Fucα1-2Galβ1-3GalNAcα-Sp8	5093	984	18
H-type4	Fucα1-2Galβ1-3GalNAcβ1-3Galα-Sp9	2119	358	14
H-type4	Fucα1-2Galβ1-3GalNAcβ1-3Galα1-4Galβ1-4Glcβ-Sp9	12579	2452	169
H-type5	Fucα1-2Galβ1-4Glcβ-Sp0	6580	1726	185
A-tri	GalNAcα1-3(Fucα1-2)Galβ-Sp8	5986	1033	66
A-tri	GalNAcα1-3(Fucα1-2)Galβ-Sp18	6016	1079	71
A-type1	GalNAcα1-3(Fucα1-2)Galβ1-3GlcNAcβ-Sp0	14	4	2
A-type2	GalNAcα1-3(Fucα1-2)Galβ1-4GlcNAcβ-Sp0	3541	651	11
A-type2	GalNAcα1-3(Fucα1-2)Galβ1-4GlcNAcβ-Sp8	2581	529	5
A-type2	GalNAcα1-3(Fucα1-2)Galβ1-4GlcNAcβ1-3Galβ1-4GlcNAcβ-Sp0	7514	1318	93
A-type2	GalNAcα1-3(Fucα1-2)Galβ1-4GlcNAcβ1-3Galβ1-4GlcNAcβ1-3Galβ1-4GlcNAcβ-Sp0	5471	827	35
A-type5	GalNAcα1-3(Fucα1-2)Galβ1-4Glcβ-Sp0	2452	487	10
A-LewisY	GalNAcα1-3(Fucα1-2)Galβ1-4(Fucα1-3)GlcNAcβ-Sp0	2348	434	16
B-tri	Galα1-3(Fucα1-2)Galβ-Sp8	2419	595	4
B-tri	Galα1-3(Fucα1-2)Galβ-Sp18	2447	564	17
B-type1	Galα1-3(Fucα1-2)Galβ1-3GlcNAcβ-Sp0	1127	177	5
B-type1	Galα1-3(Fucα1-2)Galβ1-3GlcNAcβ-Sp8	761	67	2
B-type2	Galα1-3(Fucα1-2)Galβ1-4GlcNAc-Sp0	2073	443	5
B-type3	Galα1-3(Fucα1-2)Galβ1-3GalNAcα-Sp8	65	8	4
B-type4	Galα1-3(Fucα1-2)Galβ1-3GalNAcb-Sp8	83	6	9
B-type5	Galα1-3(Fucα1-2)Galβ1-4Glcβ-Sp0	638	178	4
B-LewisY	Galα1-3(Fucα1-2)Galβ1-4(Fucα1-3)GlcNAcβ-Sp0	2172	206	13
B-LewisY	Galα1-3(Fucα1-2)Galβ1-4(Fucα1-3)GlcNAcβ-Sp8	2972	460	17
Lewisa	Galβ1-3(Fucα1-4)GlcNAcβ1-3Galβ1-4GlcNAcβ-Sp0	7682	1192	60
Lewisa	Galβ1-3(Fucα1-4)GlcNAcβ-Sp0	15130	3296	228
Lewisa	Galβ1-3(Fucα1-4)GlcNAcβ-Sp8	12765	2398	166
Sialyl_LewisA	Neu5Acα2-3Galβ1-3(Fucα1-4)GlcNAcβ-Sp8	23197	5119	430
Lewisa_sulfo	[3OSO3]Galβ1-3(Fucα1-4)GlcNAcβ-Sp8	15792	4167	405
LewisB	Fucα1-2Galβ1-3(Fucα1-4)GlcNAcβ-Sp8	7499	1291	95
LewisB	Fucα1-2Galβ1-3(Fucα1-4)GlcNAcβ1-3(Galβ1-4GlcNAcβ1-6)Galβ1-4Glc-Sp21	4620	973	50
LewisX	Galβ1-4(Fucα1-3)GlcNAcβ-Sp0	3491	678	23
LewisX	Galβ1-4(Fucα1-3)GlcNAcβ-Sp8	3883	580	27
LewisX	Galβ1-4(Fucα1-3)GlcNAcβ1-4Galβ1-4(Fucα1-3)GlcNAcβ-Sp0	6346	1156	63
LewisX	Galβ1-3GlcNAcβ1-3(Galβ1-4(Fucα1-3)GlcNAcβ1-6)Galβ1-4Glc-Sp21	3016	575	21
Sialyl_lewisX	Neu5Acα2-3Galβ1-4(Fucα1-3)GlcNAcβ-Sp0	2728	405	21
Sialyl_lewisX	Neu5Acα2-3Galβ1-4(Fucα1-3)GlcNAcβ-Sp8	3899	652	25
Sialyl_lewisX	Neu5Acα2-3Galβ1-4(Fucα1-3)GlcNAcβ1-3Galβ-Sp8	2178	636	9
Sialyl_lewisX	Neu5Acα2-3Galβ1-4(Fucα1-3)GlcNAcβ1-3Galβ1-4GlcNAcβ-Sp8	3911	662	26
LewisX_sulfo	[3OSO3]Galβ1-4(Fucα1-3)GlcNAc-Sp0	5712	1010	111
LewisX_sulfo	[3OSO3]Galβ1-4(Fucα1-3)GlcNAcβ-Sp8	6193	1088	49
LewisX_sulfo	Galβ1-4(Fucα1-3)[6OSO3]GlcNAc-Sp0	3588	728	26
LewisY	Fucα1-2Galβ1-4(Fucα1-3)GlcNAcβ-Sp0	7491	1265	76
LewisY	Fucα1-2Galβ1-4(Fucα1-3)GlcNAcβ-Sp8	6219	1063	27
