# Supplementary material for: Deciphering the Glycan Preference of Bacterial Lectins by Glycan Array and Molecular Docking with Validation by Microcalorimetry and Crystallography
Source: PLoS One. 2013 Aug 19;8(8):e71149. doi: 10.1371/journal.pone.0071149 (PMC3747263; doi:10.1371/journal.pone.0071149)
Supplement: Table S4 — Binding intensities (FU) for labeled BambL protein with glycan array chips v4.1 from the consortium for functional glycomics. Full data is available on the web site (http://www.functionalglycomics.org/). (PDF) [file pone.0071149.s007.pdf]

Table S4: Binding intensities (FU) for labeled BambL protein with glycan array chips v4.1 from the consortium for functional glycomics. Full data is available on the web site (http://www.functionalglycomics.org/)

		10 µg/ml	1 µg/ml	0.1 µg/ml
	Glycan Structure	Average	Average	Average
H-di	Fucα1-2Galβ-Sp8	1	5	3
H_type1	Fucα1-2Galβ1-3GlcNAcβ1-3Galβ1-4Glcβ-Sp8	2321	1027	683
H-type1	Fucα1-2Galβ1-3GlcNAcβ1-3Galβ1-4Glcβ-Sp10	1526	1999	186
H-type1	Fucα1-2Galβ1-3GlcNAcβ-Sp0	2419	1818	919
H-type1	Fucα1-2Galβ1-3GlcNAcβ-Sp8	13063	9008	3686
H-type1	Fucα1-2Galβ1-3GlcNAcβ1-3(Galβ1-4GlcNAcβ1-6)Galβ1-4Glc-Sp21	970	155	126
H-type2	Fucα1-2Galβ1-4GlcNAcβ1-3Galβ1-4GlcNAcβ-Sp0	6	1	0
H-type2	Fucα1-2Galβ1-4GlcNAcβ1-3Galβ1-4GlcNAcβ1-3Galβ1-4GlcNAcβ-Sp0	3	9	5
H-type2	Fucα1-2Galβ1-4GlcNAcβ-Sp0	4	2	3
H-type2	Fucα1-2Galβ1-4GlcNAcβ-Sp8	-1	4	2
H-type3	Fucα1-2Galβ1-3GalNAcα-Sp8	3927	2990	1303
H-type4	Fucα1-2Galβ1-3GalNAcβ1-3Galα-Sp9	342	48	40
H-type4	Fucα1-2Galβ1-3GalNAcβ1-3Galα1-4Galβ1-4Glcβ-Sp9	9549	6167	4179
H-type5	Fucα1-2Galβ1-4Glcβ-Sp0	4	4	5
A-tri	GalNAcα1-3(Fucα1-2)Galβ-Sp8	5	8	3
A-tri	GalNAcα1-3(Fucα1-2)Galβ-Sp18	2	2	3
A-type1	GalNAcα1-3(Fucα1-2)Galβ1-3GlcNAcβ-Sp0	2	6	5
A-type2	GalNAcα1-3(Fucα1-2)Galβ1-4GlcNAcβ-Sp0	0	10	0
A-type2	GalNAcα1-3(Fucα1-2)Galβ1-4GlcNAcβ-Sp8	3	2	1
A-type2	GalNAcα1-3(Fucα1-2)Galβ1-4GlcNAcβ1-3Galβ1-4GlcNAcβ-Sp0	1	7	4
A-type2	GalNAcα1-3(Fucα1-2)Galβ1-4GlcNAcβ1-3Galβ1-4GlcNAcβ1-3Galβ1-4GlcNAcβ-Sp0	1	9	0
A-type5	GalNAcα1-3(Fucα1-2)Galβ1-4Glcβ-Sp0	2	-2	13
A-LewisY	GalNAcα1-3(Fucα1-2)Galβ1-4(Fucα1-3)GlcNAcβ-Sp0	2	7	5
B-tri	Galα1-3(Fucα1-2)Galβ-Sp8	5	8	4
B-tri	Galα1-3(Fucα1-2)Galβ-Sp18	3	2	4
B-type1	Galα1-3(Fucα1-2)Galβ1-3GlcNAcβ-Sp0	7	3	3
B-type1	Galα1-3(Fucα1-2)Galβ1-3GlcNAcβ-Sp8	1122	517	29
B-type2	Galα1-3(Fucα1-2)Galβ1-4GlcNAc-Sp0	6	12	5
B-type3	Galα1-3(Fucα1-2)Galβ1-3GalNAcα-Sp8	37	5	1
B-type4	Galα1-3(Fucα1-2)Galβ1-3GalNAcb-Sp8	77	1205	292
B-type5	Galα1-3(Fucα1-2)Galβ1-4Glcβ-Sp0	2	2	2
B-LewisY	Galα1-3(Fucα1-2)Galβ1-4(Fucα1-3)GlcNAcβ-Sp0	6	15	8
B-LewisY	Galα1-3(Fucα1-2)Galβ1-4(Fucα1-3)GlcNAcβ-Sp8	389	52	5
Lewisa	Galβ1-3(Fucα1-4)GlcNAcβ1-3Galβ1-4GlcNAcβ-Sp0	0	-2	3
Lewisa	Galβ1-3(Fucα1-4)GlcNAcβ-Sp0	2	9	-5
Lewisa	Galβ1-3(Fucα1-4)GlcNAcβ-Sp8	3	1	3
Sialyl_LewisA	Neu5Acα2-3Galβ1-3(Fucα1-4)GlcNAcβ-Sp8	4	9	2
Lewisa_sulfo	[3OSO3]Galβ1-3(Fucα1-4)GlcNAcβ-Sp8	5	6	-1
LewisB	Fucα1-2Galβ1-3(Fucα1-4)GlcNAcβ-Sp8	25753	13533	6647
LewisB	Fucα1-2Galβ1-3(Fucα1-4)GlcNAcβ1-3(Galβ1-4GlcNAcβ1-6)Galβ1-4Glc-Sp21	13453	10217	5405
LewisX	Galβ1-4(Fucα1-3)GlcNAcβ-Sp0	3	4	-2
LewisX	Galβ1-4(Fucα1-3)GlcNAcβ-Sp8	3	5	0
LewisX	Galβ1-4(Fucα1-3)GlcNAcβ1-4Galβ1-4(Fucα1-3)GlcNAcβ-Sp0	1	-5	3
LewisX	Galβ1-3GlcNAcβ1-3(Galβ1-4(Fucα1-3)GlcNAcβ1-6)Galβ1-4Glc-Sp21	6	1	4
Sialyl_lewisX	Neu5Acα2-3Galβ1-4(Fucα1-3)GlcNAcβ-Sp0	3	18	-2
Sialyl_lewisX	Neu5Acα2-3Galβ1-4(Fucα1-3)GlcNAcβ-Sp8	4	2	3
Sialyl_lewisX	Neu5Acα2-3Galβ1-4(Fucα1-3)GlcNAcβ1-3Galβ-Sp8	5	3	0
Sialyl_lewisX	Neu5Acα2-3Galβ1-4(Fucα1-3)GlcNAcβ1-3Galβ1-4GlcNAcβ-Sp8	9	16	5
LewisX_sulfo	[3OSO3]Galβ1-4(Fucα1-3)GlcNAc-Sp0	14	0	3
LewisX_sulfo	[3OSO3]Galβ1-4(Fucα1-3)GlcNAcβ-Sp8	10	8	9
LewisX_sulfo	Galβ1-4(Fucα1-3)[6OSO3]GlcNAc-Sp0	4	19	3
LewisY	Fucα1-2Galβ1-4(Fucα1-3)GlcNAcβ-Sp0	1407	435	91
LewisY	Fucα1-2Galβ1-4(Fucα1-3)GlcNAcβ-Sp8	1632	2097	287
